# Deletion of the EphA2 receptor exacerbates myocardial injury and the progression of ischemic cardiomyopathy

**DOI:** 10.3389/fphys.2014.00132

**Published:** 2014-04-24

**Authors:** Wesley T. O'Neal, William F. Griffin, Susan D. Kent, Filza Faiz, Jonathan Hodges, Jackson Vuncannon, Jitka A. I. Virag

**Affiliations:** ^1^Department of Internal Medicine, Wake Forest School of MedicineWinston-Salem, NC, USA; ^2^Department of Physiology, Brody School of Medicine, East Carolina UniversityGreenville, NC, USA

**Keywords:** myocardial infarction, EphA2, inflammation, remodeling, ischemia

## Abstract

EphrinA1-EphA-receptor signaling is protective during myocardial infarction (MI). The EphA2-receptor (EphA2-R) potentially mediates cardiomyocyte survival. To determine the role of the EphA2-R in acute non-reperfused myocardial injury *in vivo*, infarct size, inflammatory cell density, NF-κB, p-AKT/Akt, and MMP-2 protein levels, and changes in ephrinA1/EphA2-R gene expression profile were assessed 4 days post-MI in B6129 wild-type (WT) and EphA2-R-mutant (EphA2-R-M) mice lacking a functional EphA2-R. Fibrosis, capillary density, morphometry of left ventricular chamber and infarct dimensions, and cardiac function also were measured 4 weeks post-MI to determine the extent of ventricular remodeling. EphA2-R-M infarct size and area of residual necrosis were 31.7% and 113% greater than WT hearts, respectively. Neutrophil and macrophage infiltration were increased by 46% and 84% in EphA2-R-M hearts compared with WT, respectively. NF-κB protein expression was 1.9-fold greater in EphA2-R-M hearts at baseline and 56% less NF-κB after infarction compared with WT. EphA6 gene expression was 2.5-fold higher at baseline and increased 9.8-fold 4 days post-MI in EphA2-R-M hearts compared with WT. EphrinA1 gene expression in EphA2-R-M hearts was unchanged at baseline and decreased by 42% 4 days post-MI compared with WT hearts. EphA2-R-M hearts had 66.7% less expression of total Akt protein and 59% less p-Akt protein than WT hearts post-MI. EphA2-R-M hearts 4 weeks post-MI had increased chamber dilation and interstitial fibrosis and decreased MMP-2 expression and capillary density compared with WT. In conclusion, the EphA2-R is necessary to appropriately modulate the inflammatory response and severity of early injury during acute MI, thereby influencing the progression of ischemic cardiomyopathy.

## Introduction

Myocardial Infarction (MI) is associated with an increased risk of congestive heart failure. After the initial infarction, structural remodeling ensues, leading to progressive ventricular dilation and dysfunction (Kannel, 2000; Frangogiannis et al., 2002; Jugdutt, 2012). Manipulation of cell signaling represents a new therapeutic approach to combat the development of heart failure by reducing the severity of the original ischemic insult (French et al., 2010; Kung et al., 2011).

The Eph receptors (Eph-R) and their ligands, the ephrins, are the largest family of receptor tyrosine kinases. Ephrin-Eph-R signaling regulates cell differentiation, proliferation, and migration during development (Kullander and Klein, 2002; Pasquale, 2008) and plays a pivotal role in various cancers (Brantley et al., 2002; McCarron et al., 2010). EphrinA1, an angiogenic protein stimulated by pro-inflammatory cytokines, promotes tumorigenesis through interaction with the EphA2-R (Pandey et al., 1995; Cheng et al., 2002; Brantley-Sieders et al., 2004a,b, 2006; Pasquale, 2008; Miao et al., 2009; Beauchamp and Debinski, 2011). Antagonism of the EphA2-R with lithocholic acid blocks EphA2-R phosphorylation and reduces cancer cell growth (Incerti et al., 2013). Furthermore, the absence of the EphA2-R in mice confers a predilection for hyper-responsiveness to allergens demonstrated by increased inflammation (Okazaki et al., 2009). Conversely, EphA2-R in other tissues has been demonstrated to enhance inflammation via endothelial cell priming and inflammatory cell migration (Ivanov and Romanovsky, 2006; Coulthard et al., 2012; Funk et al., 2012; Funk and Orr, 2013). EphA2-R expression on human monocytes and coronary artery endothelial cells, both of which express a combination of other ephrinA ligands and EphA receptors, may modulate inflammatory cell transmigration (Sakamoto et al., 2011). Also, EphA2-R is expressed on rat vascular smooth muscle cells and contributes to ephrinA1-mediated vessel destabilization during revascularization (Deroanne et al., 2003). The tissue- and cell-specific role of EphA2-R and its interaction with other EphA-Rs and ephrinA ligands in myocardial injury is complex and warrants investigation.

Recently, our lab and others have reported that ephrinA1-EphA-R signaling can be manipulated to preserve cardiomyocyte function after MI. We have shown that ephrinA1-Fc administration is cardioprotective during MI and possibly this, at least in part, is mediated through the EphA2-R (Dries et al., 2011). Goichberg et al. has shown that ephrinA1-Fc treatment during MI is cardioprotective through EphA2-R-mediated revascularization and regeneration (Goichberg et al., 2011). Jehle et al. demonstrated that immortalized HL-1 cardiomyocytes exposed to doxazocin were protected from apoptosis by increased EphA2-R expression and reduced phosphorylation in response to lithocholic acid (Jehle et al., 2012). Combined with its known pro-angiogenic and pro-inflammatory actions in the heart, we hypothesized that MI in mice lacking a functional EphA2-R worsens cardiomyocyte injury and inflammation leading to adverse remodeling and cardiac dysfunction (O'Neal et al., 2013). To investigate this, we examined the acute response to non-reperfused MI 4 days post-MI and the progression of ischemic cardiomyopathy 4 weeks post-MI in EphA2-R-mutant (EphA2-R-M) mice.

## Methods

### Animals

Experimental research protocols were approved by the East Carolina University Institutional Animal Care and Use Committee following the guidelines of the National Institutes of Health for the Care and Use of Laboratory Animals. B6129SF2/J mice (stock #101045) (WT) and B6129S6-Epha2^tm1Jrui^/J (stock #006028; an EphA2-deficient homozygous mutant (truncated, non-functional protein) (Brantley-Sieders et al., 2004a,b); EphA2-R-M) mice were purchased from Jackson Laboratories. All mice were housed in ventilated cages and animal care was maintained by the Department of Comparative Medicine at The Brody School of Medicine, East Carolina University. Mice were exposed to 12/12 hour light/dark cycle conditions and received food and water *ad-libitum*.

### Surgical procedure and tissue collection

Male WT and EphA2-R-M mice (8–12 weeks) were anesthetized with an intraperitoneal injection of 20 μl/g body weight Avertin (20 mg/ml) and mechanically ventilated. The left anterior descending coronary artery was permanently occluded using an 8–0 suture. The rib cage, muscle, and skin were then closed with a 6–0 suture. Four days or 4 weeks after surgery, mice were anesthetized with a lethal intraperitoneal injection of 0.1 mL pentobarbital (390 mg/mL). At the time of sacrifice, the heart was arrested in diastole using cold KCl (30 mM), excised, rinsed in saline, and immersed in zinc fixative or snap frozen in liquid nitrogen. For immunohistochemical staining and morphometric measurements, whole hearts were transversely sectioned into 4 slices of equal thickness and processed in an automated tissue processor (TP1020, Leica, Nußloch, Germany), and embedded in paraffin (Microm EC350, Richard-Allan Scientific, Kalamazoo, MI, USA) (Beckstead, 1994; Ismail et al., 2003). Five μm thick sections were mounted on Superfrost Plus glass slides for histology or immunostaining.

### Morphometry and histology

Morphometric measurements were performed blindly on tissue sections 4 days and 4 weeks post-MI. Four H&E-stained (hematoxylin and eosin) section images of each heart were taken at 20x magnification using a DP70 digital camera. Left ventricular (LV) cross-sectional area was measured using Scion imaging software (Scion Corporation, Frederick, MD, USA). LV anterior wall and septal thickness were measured using the average of 4 measurements in each of the 4 sections. The area of infarction was measured 4 days post-MI and taken as an average of total necrotic and granulation tissue areas. Infarct size (IS) was measured and reported as a percentage of the left ventricle.

Myocyte cross-sectional area (MCSA) was measured in 3–8 cardiomyocytes with centrally located nuclei in each of 6 images (600x) in the endocardium and epicardium (*n* = 3–5/group) using Scion imaging software (Scion Corporation, Frederick, MD, USA).

Interstitial fibrosis was measured using a Picrosirus Red/Fast Green staining protocol. Briefly, samples were de-paraffinized and rehydrated, immersed in a 0.1% picrosirius red/fast green solution (Sigma-Aldrich, 365548, F7258, P6744) for 30 minutes, cleared, and coverslipped (Virag et al., 2007). Tissue collagen was determined by taking 4 images at 400× in 2 sections per heart and using Adobe Photoshop CS4 (Adobe Systems, Mountview, CA) to count the number of red and green pixels. The percent collagen was calculated as the number of red pixels/red + green pixels × 100%. The numbers were averaged for each animal and the data presented are the average of 3–5 animals per group.

### Immunostaining

Tissue sections were deparaffinized in xylene and endogenous peroxidases quenched with 3% H_2_O_2_ in methanol. Slides were rinsed in saline and incubated with antibodies to CD45 (BD Biosciences; #550539) for leukocyte infiltration, Ly6G (BD Biosciences, #550291) for neutrophil infiltration, or CD31 (BD Biosciences, #553371) for capillary density. For EphA6-R staining, anti-EphA6-R (SantaCruz #25740) was used. Slides were incubated with appropriate biotinylated secondary antibodies and then with Avidin Biotin Complex (Vector Labs PK-6100). The reaction product was visualized with DAB (Vector, SK-4100), counterstained with methyl green, dehydrated in xylene, and slides were coverslipped.

### qRT-PCR

Whole left ventricles of uninjured (baseline) hearts and hearts 4 days post-MI from WT and EphA2-R-M mice were homogenized using Trizol for RNA isolation. Purification was performed using the Qiagen RNeasy kit. cDNA was made for each sample using a high capacity cDNA kit. Real-time PCR (qRT-PCR) was performed using an Applied Biosystems thermocycler. TaqMan primers were obtained from Applied Biosciences (ephrinA1: Mm00438660_m1, EphA1: Mm00445804_m1, EphA2: Mm00438726_m1, EphA3: Mm00580743_m1, EphA4: Mm00433056_m1, EphA5: Mm00433074_m1, EphA6: Mm00433094_m1, EphA7: Mm00833876_m1, GAPDH: Mm99999915_g1). All samples were run in triplicate and a reaction mixture of 10 μ l (100 ng RNA) was amplified using recommended conditions. Gene expression was normalized to glyceraldehyde-3-phosphate dehydrogenase (GAPDH) expression. Fluorescence data were analyzed using the ΔΔ Ct method.

### Western blotting

Whole left ventricles of baseline hearts and hearts 4 days post-MI from WT and EphA2-R-M mice were homogenized in a lysis buffer containing 50 mM Hepes, 10 mM EDTA, 100 mM NaF, 50 mM sodium pyrophosphate, 1% protease, and 1% phosphatase inhibitors. The Bradford Assay was used to quantify the amount of protein. Western blotting was performed on a 4–12% gradient Bis-Tris gel (BioRad) in 1 × Mops running buffer.

Fifty micrograms of sample was loaded per well. The gel was run for 1 h at 155 V, and transferred onto pure nitrocellulose membranes (BioRad). Antibodies: GAPDH (Millipore, #MAB374), p-Akt (Cell Signaling, #4060), Akt (Cell Signaling, #4691), matrix metalloproteinase-2 (MMP-2; R&D Systems, #AF1488), and NF-κBp65 (Santa Cruz, #sc-372) followed by appropriate secondary antibodies. All blots were detected with Amersham ECL Advance (GE Healthcare) and imaged on a Typhoon Imager. Densitometry was performed using Image J software (v1.42, NIH, Bethesda, MD) and the intensity of each protein was normalized to GAPDH.

### Echocardiography

Echocardiography was performed on conscious uninjured and infarcted mice at 4 weeks post-MI mice after they had been acclimated in 2–3 sessions within 3 days prior to data acquisition (Yang et al., 1999). A VisualSonics Vevo 2100 diagnostic ultrasound, using M-mode, was used to obtain LV dimensions in diastole and systole. End-diastolic measurements (IVSd, LVPWd, and LVIDd) were obtained at the point of maximal LV diastolic dimension. End-systolic dimensions (IVSs, LVPWs, and LVIDs) were measured at the time of most anterior systolic excursion of the LVPW associated with minimal chamber dimension. Average measurements were calculated using the leading-edge technique of 3- to 5-consecutive sinus beats. Ejection fraction (EF) was calculated from LV dimensions above using the following formula: (LVIDd^3^-LVIDs^3^)/LVIDd^3^ × 100%.

### Statistics

ANOVA (analysis of variance) with Student-Newman Keuls multiple comparison *post-hoc* analysis illustrated which groups were statistically significantly different, with significance of at least *p* < 0.05.

## Results

### Baseline characteristics

WT (*n* = 6) mice and EphA2-R-M (*n* = 7) mice weighed 30.22 ± 1.0 g and 22.39 g ± 0.5, respectively (*p* < 0.001). The LV area of EphA2-R-M hearts was 40% smaller than WT hearts (*p* < 0.05).

**Figure 1:**
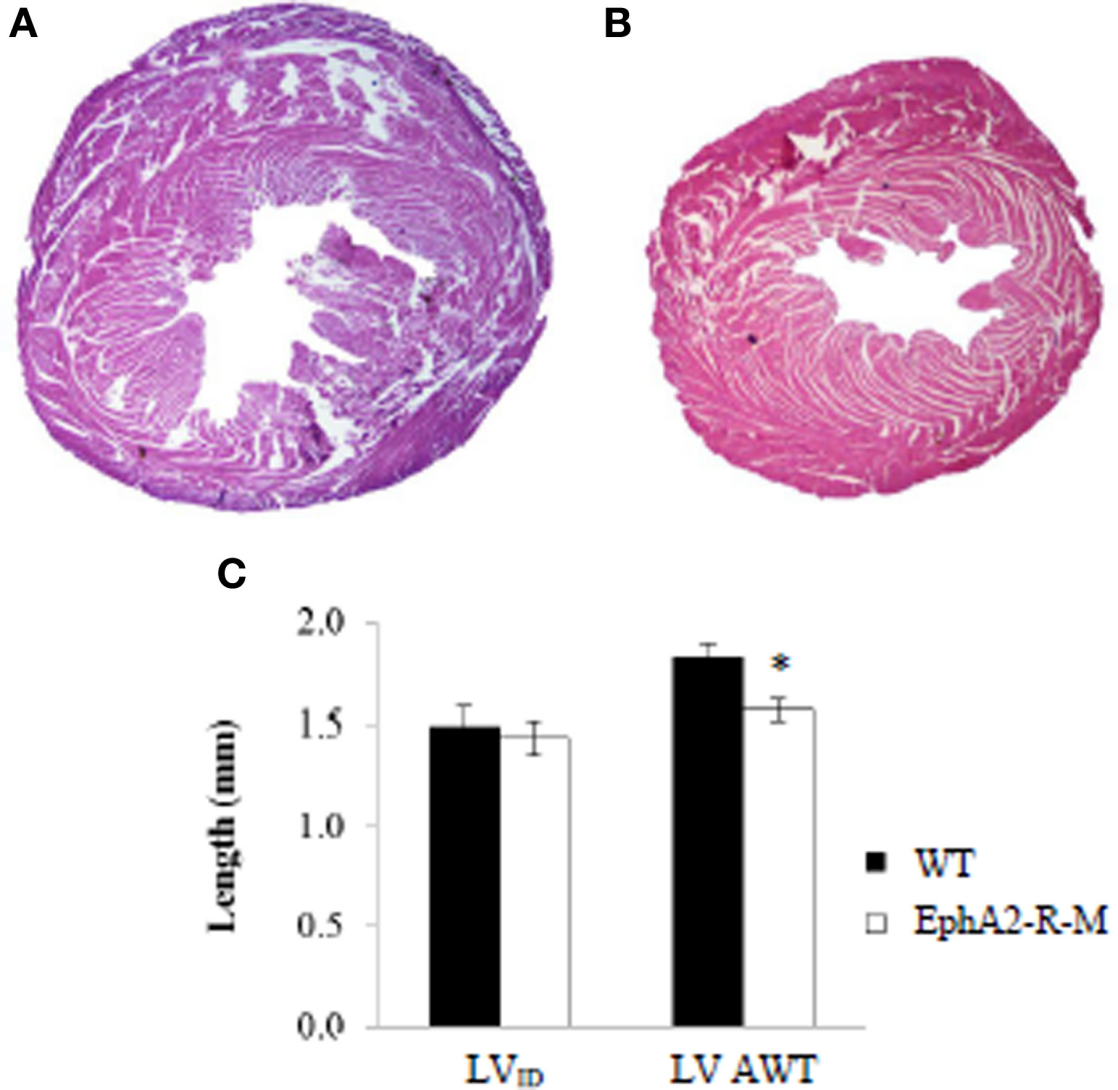
**Representative H&E Stains and Morphometry of Baseline WT and EphA2-R-M Hearts**. WT **(A)** and EphA2-R-M **(B)** hearts from baseline mice were stained with H&E (20x). The LV cross-sectional area of the EphA2-R-M hearts was 40% smaller than WT hearts (*p* < 0.05). At baseline, LV_ID_ was 1.49 ± 0.09 mm and 1.44 ± 0.08 mm in B6 WT (*n* = 6) and EphA2-R-M (*n* = 7) mice, respectively. AWT was 14% less in EphA2-R-M (*n* = 7) hearts compared with WT (*n* = 6) hearts (^*^p < 0.01) **(C)**. AWT, average wall thickness; LV, left ventricle; LV_ID_, Left ventricular internal diameter.

LV parameters, LV internal diameter (LV_ID_), and LV average wall thickness (AWT) were recorded at baseline (Figure 1). LV_ID_ was 1.49 ± 0.09 mm and 1.44 ± 0.08 mm in WT (*n* = 6) and EphA2-R-M (*n* = 7) hearts, respectively (*p* = 0.69). LV AWT in WT (*n* = 6) and EphA2-R-M (*n* = 7) hearts was 1.83 ± 0.06 and 1.57 ± 0.05 mm, respectively (*p* < 0.01). No differences in MCSA were observed (data not shown).

### Role of EphA2-R in acute injury

#### Infarct size

**Figure 2:**
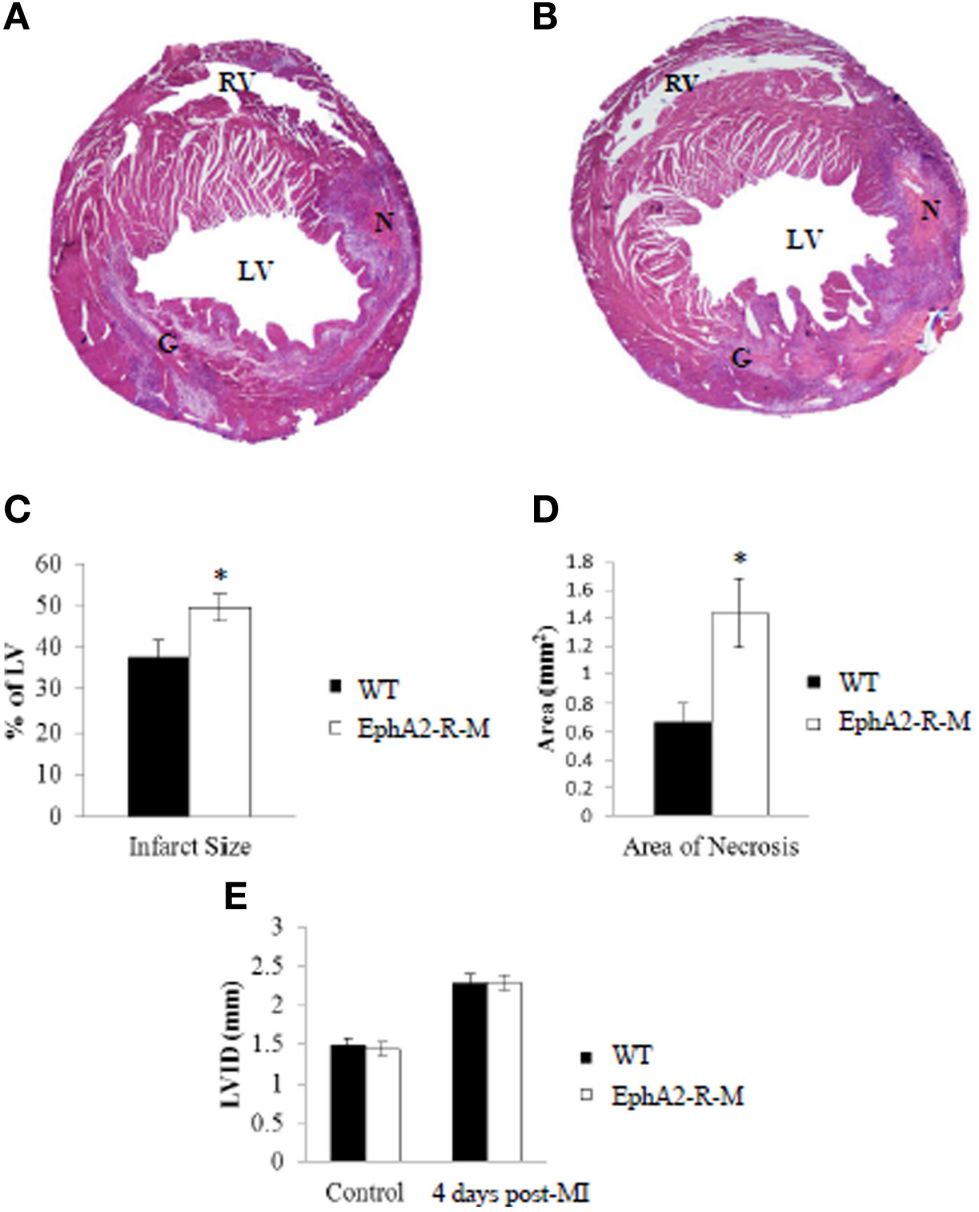
**Representative H&E Stains and Morphometry of WT and EphA2-R-M Hearts 4 Days Post-MI**. H&E Stain of WT **(A)** and EphA2-R-M **(B)** hearts 4-days post-MI. Infarct Size **(C)** was increased by 31.7% in EphA2-R-M hearts (^*^p < 0.05) and Area of Necrosis **(D)** was increased by 113% compared with WT hearts (^*^p < 0.05). LV_ID_ in EphA2-R-M hearts was increased by 59% after infarction and increased by 53% in WT hearts **(E)**. G, Granulation Tissue; LV, left ventricle; LV_ID_, Left ventricular internal diameter; N, Necrosis; RV, Right Ventricle.

IS was calculated 4 days after infarction in WT (*n* = 12) and EphA2-R-M (*n* = 9) hearts (Figure 2). IS was 37.54 ± 4.31% and 49.46 ± 3.29% in WT and EphA2-R-M hearts, respectively (*p* < 0.05). Area of necrosis was increased by 113% in EphA2-R-M hearts compared with WT (*p* < 0.05). LV_ID_ was increased by 59% and 53% in EphA2-R-M (*n* = 12) and WT (*n* = 10) hearts (NS), respectively. No difference in LV AWT was observed in EphA2-R-M (1.34 ± 0.05 mm) hearts compared with WT (1.31 ± 0.06 mm). There were no differences in MCSA (data not shown).

#### Ly6-G^+^ neutrophil density

**Figure 3:**
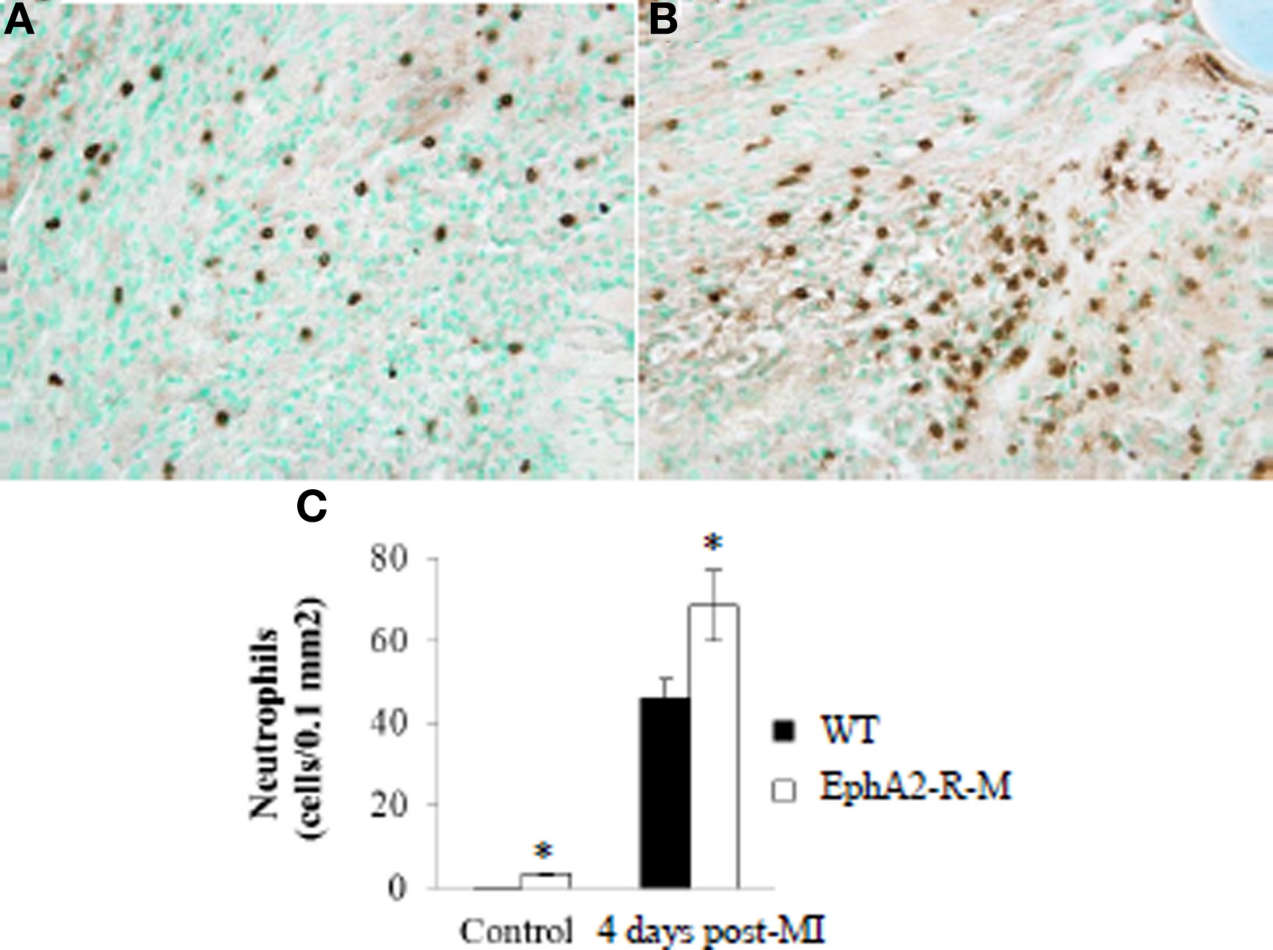
**Neutrophil Infiltration in WT and EphA2-R-M Hearts at Baseline and 4 Days Post-MI**. Ly6-G^+^ neutrophil infiltration 4 days Post-MI at 40x magnification in WT **(A)** and EphA2-R-M **(B)** hearts. Neutrophil infiltration was increased by 46% in EphA2-R-M hearts (**C**; ^*^p < 0.05).

Neutrophil infiltration was increased in baseline EphA2-R-M mice and this difference increased by 46% after MI (Figure 3). Ly6-G^+^ staining of neutrophil density in WT (*n* = 6) hearts was 0.21 ± 0.04 cells/0.1 mm^2^ and 3.48 ± 0.29 cells/0.1 mm^2^ in EphA2-R-M (*n* = 3) hearts (*p* < 0.01). Ly6-G^+^ staining 4 days post-MI in WT (*n* = 10) hearts was 46.96 ± 4.39 cells/0.1 mm^2^ compared with 68.56 ± 8.59 cells/0.1 mm^2^ in EphA2-R-M (*n* = 9) hearts (*p* < 0.05).

#### CD45^+^ leukocyte density

**Figure 4:**
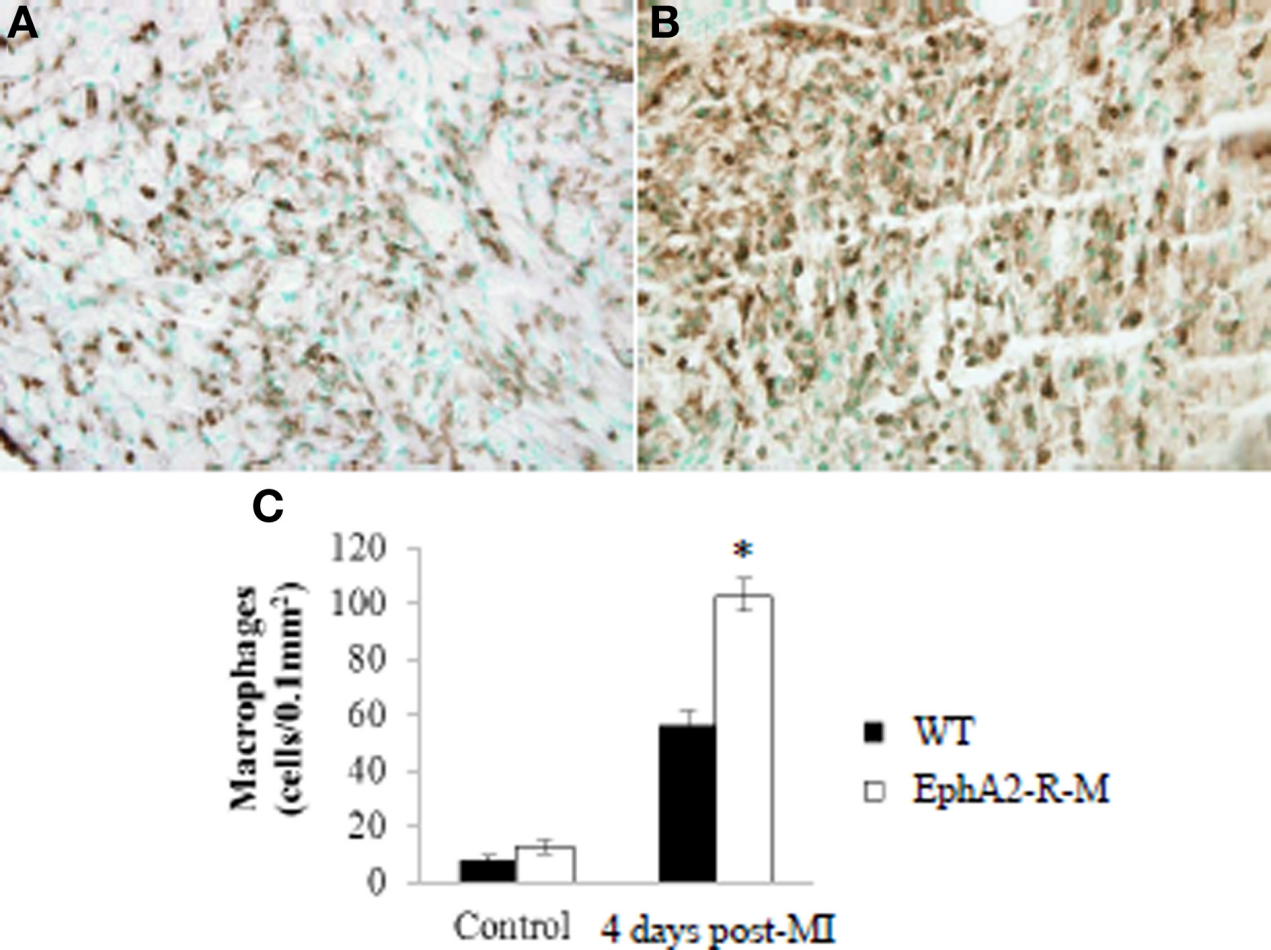
**CD45^+^ Leukocyte Density in WT and EphA2-R-M Hearts at Baseline and 4 Days Post-MI**. Leukocyte density staining 4 days post-MI at 40x magnification in WT **(A)** and EphA2-R-M **(B)** hearts. Leukocyte infiltration was increased by 84% in EphA2-R-M hearts after MI compared with WT hearts (**C**; ^*^p < 0.001).

Leukocyte density in control animals was no different between WT and EphA2-R-M hearts (Figure 4). CD45^+^ staining of leukocyte infiltration in WT (*n* = 3) mice was 7.63 ± 1.81 leukocyte/μm^2^ compared with 12.54 ± 2.41 leukocyte/μm^2^ in EphA2-R M (*n* = 10) hearts (*p* = 0.13). Leukocyte infiltration was increased by 84% in EphA2-R-M hearts after MI compared with WT hearts. CD45^+^ staining 4 days post-MI in WT (*n* = 7) and EphA2-R-M (*n* = 8) hearts was 55.97 ± 5.55 leukocyte/μm^2^ and 102.76 ± 5.94 leukocyte/μm^2^, respectively (*p* < 0.001).

#### Western blotting

**Figure 5:**
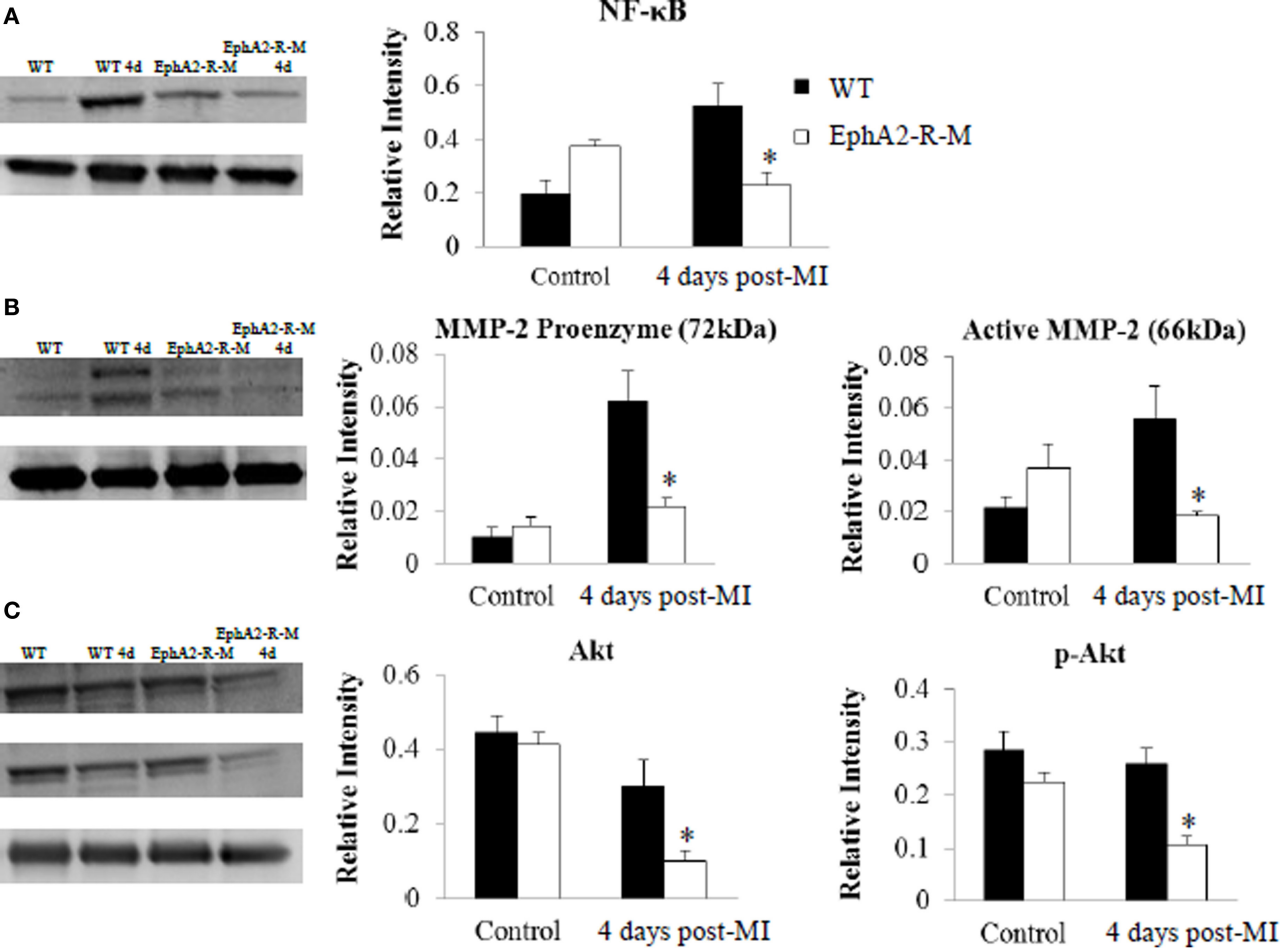
**Western Blotting of NF-κB, MMP-2, and Akt in WT, and EphA2-R-M Hearts**. NF-κB expression **(A)** was elevated nearly 3-fold in WT hearts 4 days post-MI compared with baseline (^*^p < 0.01). A similar increase was not observed in EphA2-R-M hearts compared with baseline. Both MMP-2 proenzyme and active form were 6- and 3-fold higher in WT hearts post-MI **(B)** respectively (^*^p < 0.05). No change in MMP-2 expression was observed in EphA2-R-M hearts. The expression of Akt and p-Akt was significantly decreased in EphA2-R-M hearts post-MI and it was unchanged in WT hearts (^*^p < 0.05) **(C)**.

A 2.7-fold increase in NF-κB expression was observed in WT (*n* = 3; *p* < 0.01) hearts 4 days post-MI compared with no change in EphA2-R-M (*n* = 3) hearts (Figure 5A). Both pro- and active-MMP-2 were increased 6- and 3-fold in WT mice 4 days post-MI, respectively (*p* < 0.05). No changes were observed in MMP-2 expression in EphA2-R-M hearts 4 days post-MI (Figure 5B). No differences in baseline expression of Akt or p-Akt were observed between EphA2-R-M and WT mice. EphA2-R-M hearts (*n* = 3) had 66.7% less expression of total Akt protein and 59% less p-Akt protein than WT (*n* = 3) hearts 4 days post-MI (Figure 5C).

#### qRT-PCR

**Figure 6:**
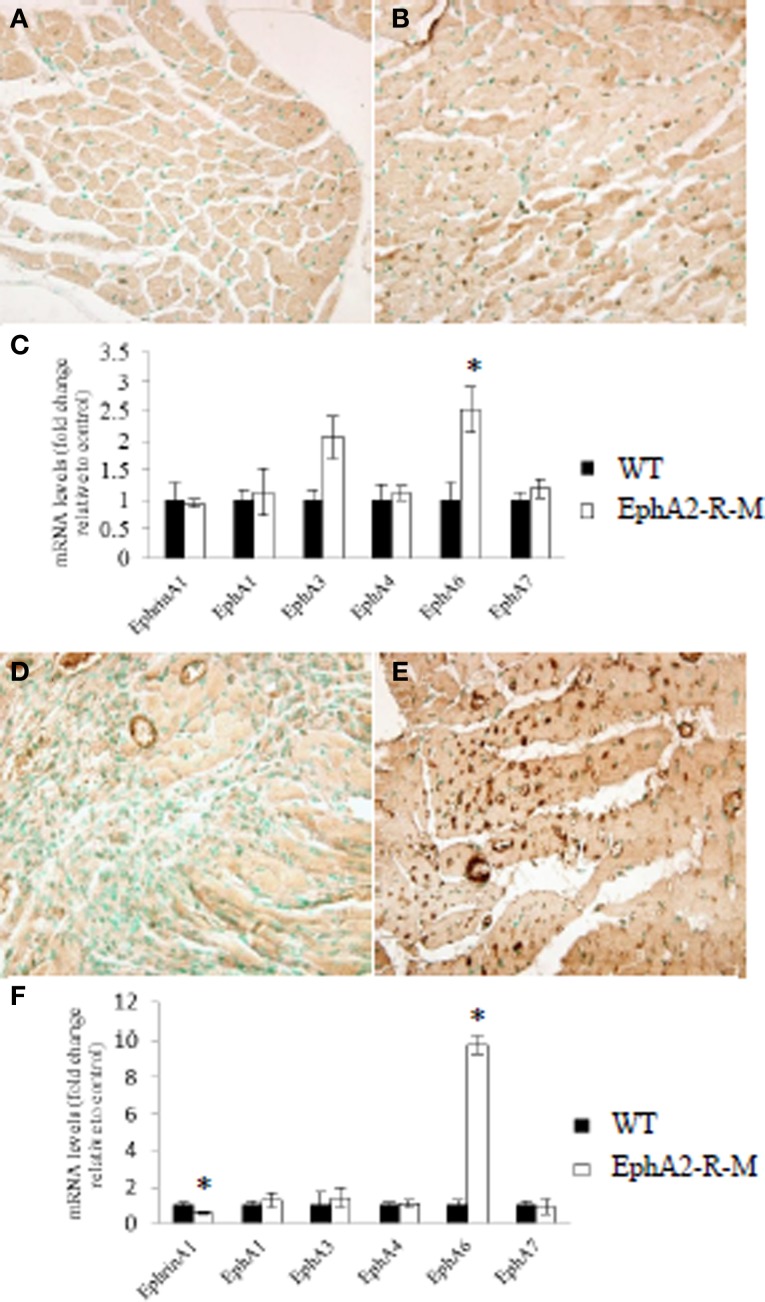
**EphA6-R Staining and EphA-R Gene Expression Profiles in WT and EphA2-R-M Hearts at Baseline and 4 Days Post-MI**. Representative immunohistochemical staining at 40x of EphA6-R in WT **(A)** and EphA2-R-M **(B)** baseline hearts and 4 days post-MI (WT: **D**; EphA2-R-M: **E**). EphrinA1 ligand and EphA-R gene expression in WT and EphA2-R-M hearts was performed in hearts at baseline **(C)** and 4 days post-MI **(F)**. In baseline hearts, EphA6-R gene expression was 2.5-fold greater in EphA2-R-M hearts (^*^p < 0.05) **(C)**. EphrinA1 ligand expression was decreased 42% (^*^p < 0.01) and EphA6-R expression was increased 9.8-fold in EphA2-R-M hearts (^*^p < 0.01) **(F)**.

EphrinA1 and EphA-R gene expression was quantified using qRT-PCR mRNA levels for baseline WT mice (*n* = 3) and EphA2-R-M mice (*n* = 3) (Figure 6C). Although ephrinA1 expression was not different between WT and EphA2-R-M mice at baseline, EphA6 gene expression was 2.5-fold greater in EphA-R-M (Figure 6B) mice compared with WT (Figure 6A) mice (*p* < 0.05). Of note, EphA3 gene expression was 2.06-fold greater in EphA2-R-M mice compared with WT; however, this difference was not statistically significantly (*p* = 0.06).

EphrinA1 and EphA-R gene expression also was quantified in WT (*n* = 3) and EphA2-R-M (*n* = 3) hearts 4 days post-MI (Figure 6F). The expression of ephrinA1 was decreased 42% in EphA2-R-M hearts compared with WT mice (*p* < 0.01). EphA6-R expression was 9.8-fold greater in EphA2-R-M (Figure 6E) mice compared with WT (Figure 6D) mice 4 days post-MI (*p* = 0.01).

#### EphA6-R immunohistochemistry

Increased expression of EphA6-R in EphA2-R-M mice was noted in mice 4 days post-MI compared with WT mice (Figure 6E). This was supported by the gene expression profile of the EphA6-R observed in EphA2-R-M mice 4 days post-MI (Figure 6F).

### Role of the EphA2-R in the progression of ischemic cardiomyopathy

#### Left-ventricular dimensions

**Figure 7:**
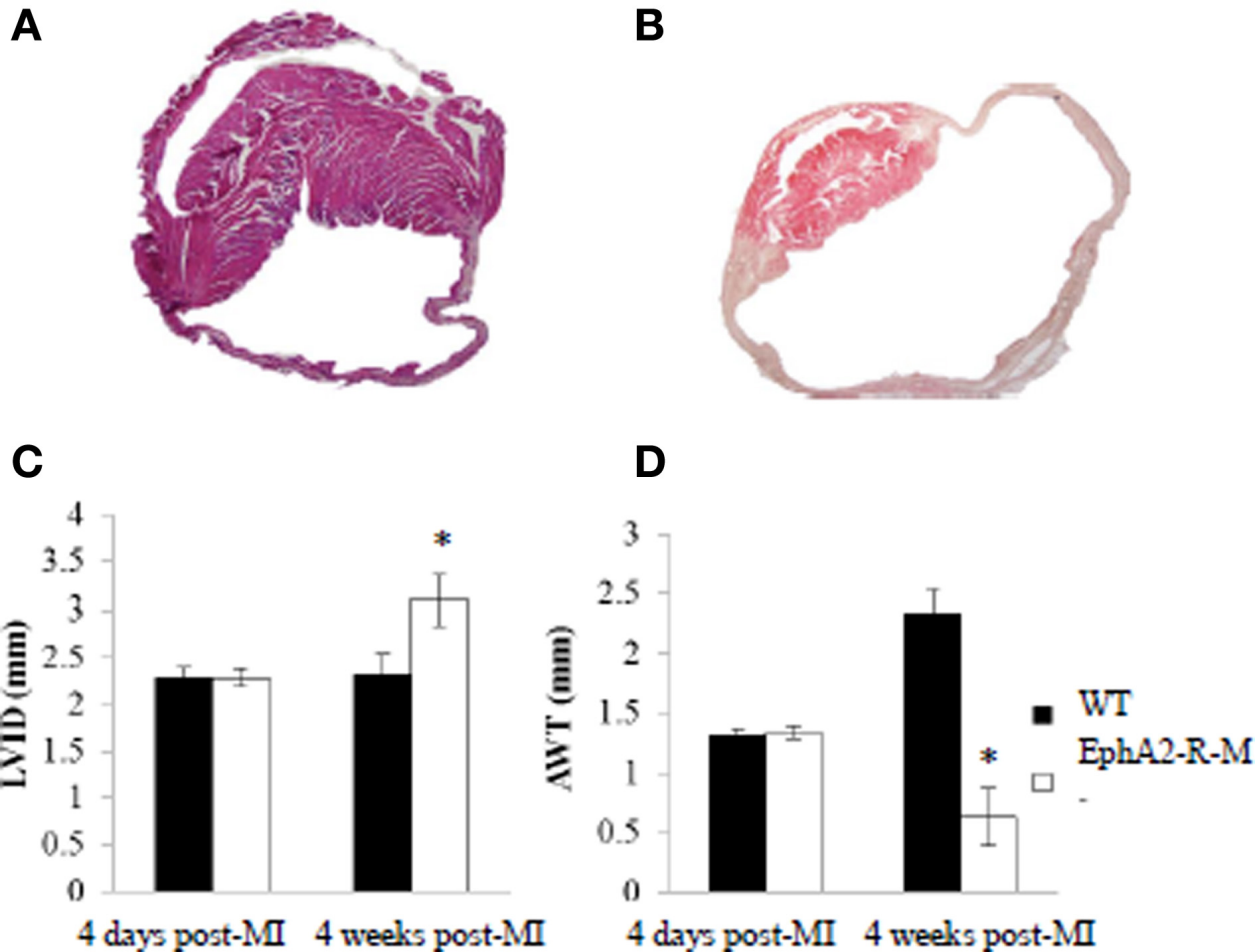
**H&E Stain of WT and EphA2-R Hearts 4 Weeks Post-MI**. WT **(A)** and EphA2-R-M **(B)** hearts 4 weeks post-MI were stained with H&E (20x). LV_ID_ increased by 35% (^*^p < 0.05) in EphA2-R-M from 4 days to 4 weeks post-MI **(C)**. In WT hearts, LV_ID_ was relatively unchanged. AWT decreased by 53% (^*^p < 0.05) in EphA2-R-M at 4 weeks compared to 4 days while AWT was increased by 78% (^*^p < 0.05) in WT 4 weeks post-MI **(D)**. LV_ID_, Left ventricular internal diameter; AWT, average wall thickness.

Images of WT and EphA2-R-M hearts 4 weeks post-MI are shown in Figure 7. EphA2-R-M hearts had a 34% increase in LV_ID_ compared with WT hearts. WT (*n* = 6) mice observed a LV_ID_ of 2.33 ± 0.21 mm and EphA2-R-M (*n* = 5) mice had a LV_ID_ of 3.10 ± 0.26 mm (*p* < 0.05). AWT in WT (*n* = 6) and EphA2-R-M (*n* = 5) mice was 1.28 ± 0.22 mm and 0.63 ± 0.24, respectively (*p* < 0.05). No differences in MCSA were observed (data not shown).

#### Fibrosis

**Figure 8:**
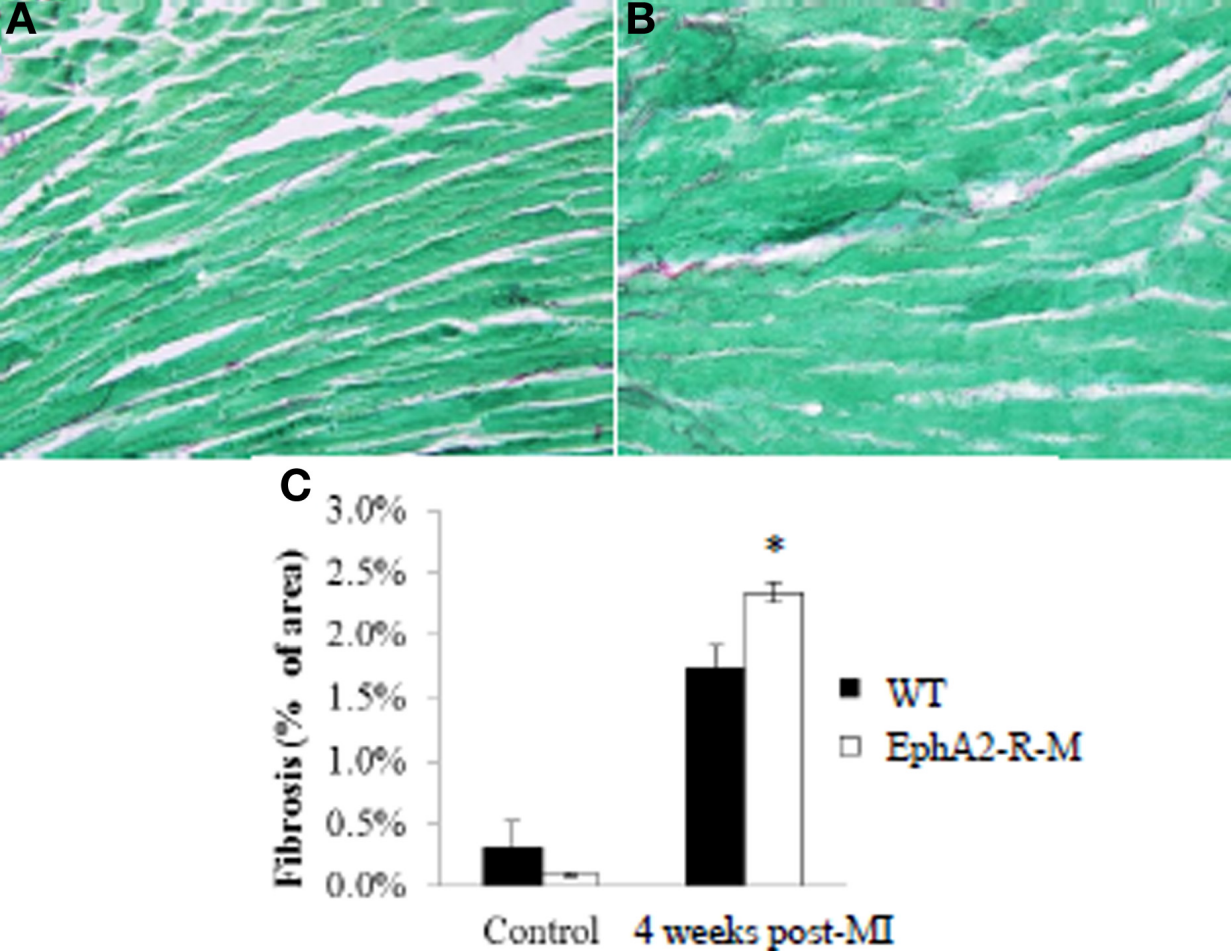
**Interstitial Fibrosis in WT and EphA2-R-M Mice 4 Weeks Post-MI**. WT **(A)** and EphA2-R-M **(B)** images depicting interstitial fibrosis 4 weeks after infarction (400x). EphA2-R-M hearts had 26% (^*^p < 0.01) more fibrosis present 4 weeks post-MI compared with WT hearts **(C)**.

Interstitial fibrosis was not different between WT and EphA2-R-M mice at baseline and 4 weeks post-MI (Figure 8). Interstitial fibrosis, presented as a percentage of the LV, in WT (*n* = 3) hearts was 0.31 ± 0.22% compared with 0.10 ± 0.02% in EphA2-R-M (*n* = 5) hearts (*p* = 0.43). There was a 26% increase (*p* < 0.01) in interstitial fibrosis in EphA2-R-M hearts (2.34 ± 0.08%) compared with WT (1.74 ± 0.18%).

#### Capillary density

**Figure 9:**
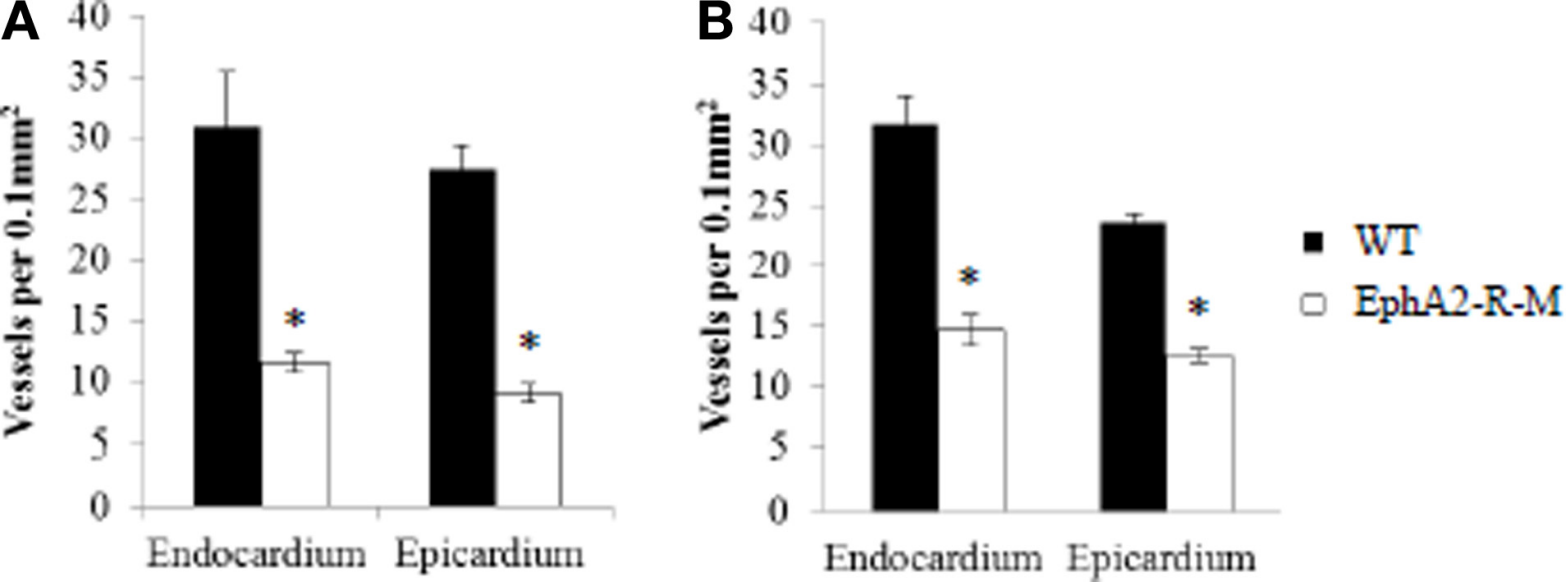
**Capillary Density of WT and EphA2-R-M Hearts 4 Days and 4 Weeks Post-MI**. Both endocardial and epicardial capillary density were significantly reduced in EphA2-R-M hearts compared with WT 4 days **(A)** and 4 weeks **(B)** post-MI (^*^p < 0.05).

Endocardial capillary density in baseline hearts was 31% less in EphA2-R-M (*n* = 5) hearts compared with WT (*n* = 6) (WT: 170 ± 20 vessels per 400x high power field; EphA2-R -M: 117 ± 7 vessels per 400x high power field; *p* = 0.04) and epicardial capillary density was decreased 24% in EphA2-R-M mice compared with WT (WT: 134 ± 15 vessels per 400x high power field; EphA2-R-M: 101 ± 12 vessels per 400x high power field; *p* < 0.05). Endocardial capillary density 4 days post-MI was reduced by 63% in EphA2-R-M hearts (Figure 9A). WT mice (*n* = 4) and EphA2-R-M (*n* = 6) endocardial capillary density was 31 ± 4 vessels per 400x high power field and 12 ± 1 vessels per 400x high power field, respectively (*p* = 0.02). In the epicardium 4 days post-MI, there were 27 ± 2 vessels per 400x high power field in WT mice (*n* = 4) and 9 ± 7 vessels per 400x high power field in EphA2-R-M mice (*n* = 6) (*p* < 0.01) (Figure 9A).

Four weeks post-MI, capillary density in the endocardium of EphA2-R-M hearts was decreased by 53% compared with WT hearts (Figure 9B). Endocardial capillary density 4 weeks post-MI in WT mice (*n* = 5) and EphA2-R-M mice (*n* = 3) was 32 ± 2 vessels per 400x high power field and 15 ± 1 vessels per 400x high power field, respectively (*p* < 0.01). In the epicardium, capillary density in EphA2-R-M hearts was reduced by 47% compared with WT (Figure 9B). WT mice (*n* = 5) had 23 ± 1 vessels per 400x high power field and EphA2-R-M mice (*n* = 3) had a capillary density of 13 ± 1 vessels per 400x high power field (*p* < 0.01).

#### Cardiac function

**Figure 10:**
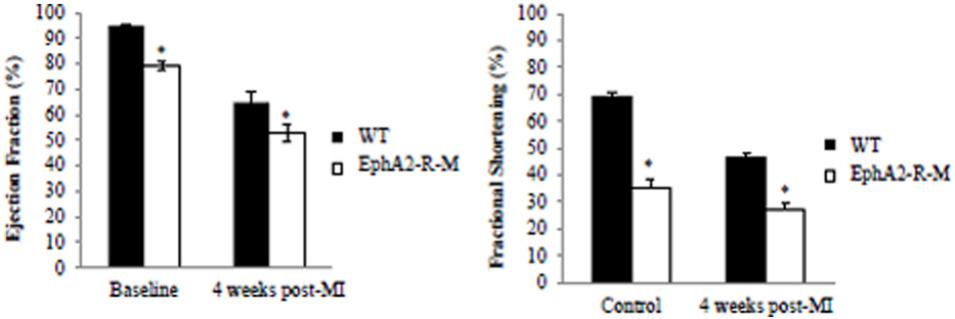
**WT and EphA2-R-M Cardiac Function at Baseline and 4 Weeks Post-MI**. Baseline EphA2-R-M mice exhibited cardiac dysfunction compared with baseline WT mice and ejection fraction and fractional shortening were depressed in both strains at 4 weeks post-MI with more significant dysfunction observed in EphA2-R-M mice at 4 weeks post-MI (Ejection Fraction: ^*^p < 0.01; Fractional Shortening: ^*^p < 0.05).

No differences in blood pressure or heart rate in conscious mice between WT and EphA2-R-M mice was observed (data not shown). Cardiac function in EphA2-R-M mice was significantly poorer than WT mice at baseline and 4 weeks post-MI (Figure 10). Baseline ejection fraction in WT (*n* = 11) mice was 94.85 ± 0.85% compared with 78.99 ± 1.51% in EphA2-R-M (*n* = 22) mice (*p* < 0.001). Ejection fraction 4 weeks post-MI in WT (*n* = 5) and EphA2-R-M (*n* = 12) mice was 64.91 ± 3.70% and 52.78 ± 3.59%, respectively (*p* = 0.04). Baseline fractional shortening in WT mice was 68.74 ± 6.6% compared with 49.72 ± 11.04% in EphA2-R-M (*p* < 0.001). At 4 weeks post-MI, fractional shortening was 35.40 ± 6.23% in WT mice and 26.89 ± 7.69% in EphA2-R-M mice (*p* < 0.05). Chamber dimensions obtained with echocardiographic imaging (data not shown) follow the same trends as those obtained with morphometric analyses.

## Discussion

This is the first report to examine the EphA2-R's role in regulating the cellular events after permanent coronary occlusion and its involvement in the progression of ischemic cardiomyopathy. Specifically, we have demonstrated that the EphA2-R exacerbates the inflammatory response and influences early cardiomyocyte injury that ultimately affects chronic ventricular remodeling.

Currently, the EphA2-R's involvement in regulating acute ischemic injury in the heart is unknown. In a model of ischemic brain injury, Thundyil et al. observed less inflammatory cell infiltrate and reduced apoptosis in EphA2-R-M mice (Thundyil et al., 2013). In contrast, EphA2-R activation protects HL-1 cardiomyocytes from apoptosis and ischemic injury in the kidney (Baldwin et al., 2006; Jehle et al., 2012). These findings provide evidence that the regulation of acute ischemia by the EphA2-R is a tissue- and cell-specific phenomenon.

EphrinA reverse signaling has been implicated in regulation of Akt phosphorylation and inhibition of apoptosis (Holen et al., 2008). Additionally, our lab has shown that the upregulation and phosphorylation of Akt protein is associated with improved cardiomyocyte survival in ephrinA1-Fc treated hearts (Brantley-Sieders et al., 2005; Dries et al., 2011). In the current study, we observed decreased expression of Akt and p-Akt in EphA2-R-M hearts 4 days post-MI compared with WT, implicating the EphA2-R's role in modulating cardioprotective Akt signaling.

Necrotic cardiomyocytes trigger an inflammatory response, involving the transmigration of neutrophils and leukocytes which clear debris and release cytokines to promote granulation tissue formation (Frangogiannis et al., 2002; Virag and Murry, 2003; Frangogiannis, 2008, 2012). Neutrophil and macrophage infiltration were significantly increased in EphA2-R-M mice 4 days post-MI while NF-κB expression was decreased. This paradoxical finding suggests that EphA2-R-M cardiomyocytes have lost their ability to regulate inflammation after infarction. Additionally, the increased residual necrosis and decreased MMP-2 synthesis and activation in EphA2-R-M hearts potentially is explained by defective inflammatory cell function resulting from deficient NF-κB expression, leading to poorer remodeling and dysfunction. The EphA2-R is a known regulator of inflammation in endothelial cells (Funk et al., 2012; Funk and Orr, 2013) and infiltration of leukocytes occurs in lung tissue deficient of this receptor (Okazaki et al., 2009). Therefore, our work provides evidence that the EphA2-R modulates inflammation in the ischemic myocardium and that defective regulation leads to an elevated inflammatory response. Further studies are needed to examine the cell-specific molecular consequences of NF-κB deficiency in EphA2-R-M mice.

Macrophages persist in the infarcted area after MI and release chemokines, such as MCP-1 and IL-1β, which recruit fibroblasts to deposit collagen to form fibrotic scar tissue. This ultimately leads to progressive ventricular dilation and dysfunction (Nian et al., 2004; Frangogiannis and Entman, 2005). Increased macrophage infiltration and subsequent collagen deposition were observed in EphA2-R-M mice. The resultant enhanced ventricular wall thinning explains the poorer functional capacity of EphA2-R-M mice. Also, a 6-fold change in MMP-2 pro-enzyme expression and a 3-fold increase in the active form were observed in WT mice 4 days post-MI. However, in EphA2-R-M mice, no significant changes in either form of MMP-2 were observed at baseline or 4 days post-MI. The EphA2-R mediates the acute inflammatory response post-MI and this ultimately influences chronic ventricular remodeling leading to ischemic cardiomyopathy. Cell-specific expression of EphA2-R in the various cells involved in matrix turnover will give further insight into this mechanism.

The EphA2-R is an established angiogenic factor that contributes to vascular development and post-natal angiogenesis. In isolated human brain endothelial cells, inhibition of the EphA2-R with siRNA has been shown to reduce angiogenesis (Zhou et al., 2011). Overexpression of the EphA2-R is associated with increased angiogenesis in ovarian cancer cells and tumorigenesis is impaired in EphA2-R deficient mice (Brantley-Sieders et al., 2005; Merritt et al., 2011). While angiogenesis in oncogenic pathologies is undesirable, similar mechanisms exist as pro-angiogenic stimuli in ischemic tissue which may present a clinical dilemma requiring careful pre-treatment evaluation. The pro-angiogenic properties of the EphA2-R in the heart and in response to ischemia have not been previously described. The capillary density in the endocardium and epicardium was significantly reduced in baseline EphA2-R-M mice and this difference persisted after infarction. This finding supports the role of EphA2-R in vascular development and post-MI neo-angiogenesis in the murine heart.

To our knowledge, the functional capacity of mice deficient of the EphA2-R has not been reported. At baseline, ejection fraction of EphA2-R-M mice was slightly but significantly reduced compared with WT animals, suggesting a developmental role of the EphA2-R in the functional capacity of the heart. The EphA2-R has been implicated in spinal neuralation (Abdul-Aziz et al., 2009). However, no literature exists on its role in cardiac development or in cardiac physiology. Decreased capillary density potentially explains the poorer function of the EphA2-R-M mice. This would result in a limited ability of the myocardium to respond to alterations in perfusion or oxygen concentration. Although we have not performed functional analyses of the vessels or determined whether this deficiency alters perfusion, it is possible that the inadequate revascularization we observed post-MI explains our findings in the EphA2-R-M myocardium.

Our previous work has reported that the expression profiles of the ephrinA1 ligand and the EphA-Rs (A1-4, 6, and 7) change in the heart after MI and with treatment of exogenous ephrinA1-Fc ligand (Dries et al., 2011). In our prior study, the EphA6-R was downregulated and the EphA2-R was reciprocally upregulated in response to MI and with ephrinA1-Fc treatment. The current study supports the reciprocal expression profile of the EphA2-R and the EphA6-R as EphA6-R expression was increased in EphA2-R-M mice. Potentially, significant cross-talk between these receptors exist to alter activation or inhibition of each receptor such that EphA6 upregulation in the EphA2-R-M offsets the deleterious effects of the absence of EphA2-R. This is supported by the fact that EphA and EphB receptors have been shown to form heterotypic clusters upon ligand binding and activation or inhibition of the signaling cascades depends on these receptors' specific interactions (Janes et al., 2011). The EphA2-R most likely mediates cardioprotection and interacts with the EphA6-R to decrease potential negative sequelae mediated through this receptor. Further studies to characterize the cell-specific expression profiles of the EphA2-R and EphA6-R are needed to elucidate their interaction and role in post-MI myocardium.

Expression of the protective ephrinA1 ligand was significantly decreased in EphA2-R-M mice after MI. This is consistent with our previous work (Dries et al., 2011). The reduction in the ephrinA1 ligand gene expression in our current study supports our previous finding that the cardioprotective benefits of endogenous ephrinA1 are lost after MI and worsened in EphA2-R-M mice. Since this study does not explore the potential of ephrinA1 treatment to reduce injury during MI, further studies are warranted to determine if the cardioprotective benefits of this ligand can be reproduced in mice that lack the EphA2-R.

In conclusion, this study has demonstrated the complex role of the EphA2-R in the heart after MI. The EphA2-R is a critical mediator of inflammation and early injury during acute MI which influences the progression of ischemic cardiomyopathy. EphrinA1-EphA signaling represents an exciting new area of exploration in the heart and further studies are needed to determine their role and therapeutic potential to blunt injury in MI.

## Author contributions

Wesley T. O'Neal and William F. Griffin compiled, analyzed, and interpreted all data and drafted the manuscript. Susan D. Kent carried out the mouse breeding, performed the RT-PCR, western blotting, histology and immunohistochemistry, and blood pressure experiments. Filza Faiz, Jonathan Hodges, and Jackson Vuncannon assisted in collecting and compiling data and editing the manuscript. Jitka A. I. Virag designed the study, obtained the funding, surgical procedures, and echocardiography, supervised data collection, analyzed and interpreted data, and edited the manuscript to ensure accuracy and inclusion of important intellectual content. All authors have read the manuscript and have given final approval of this version.

### Conflict of interest statement

The authors declare that the research was conducted in the absence of any commercial or financial relationships that could be construed as a potential conflict of interest.
